# Aminoglycoside resistance profile and structural architecture of the aminoglycoside acetyltransferase AAC(6')-Im

**DOI:** 10.15698/mic2017.12.602

**Published:** 2017-11-09

**Authors:** Clyde A. Smith, Monolekha Bhattacharya, Marta Toth, Nichole K. Stewart, Sergei B. Vakulenko

**Affiliations:** 1 Stanford Synchrotron Radiation Lightsource, SLAC National Accelerator Laboratory, Menlo Park, CA 94025, USA.; 2 Department of Chemistry and Biochemistry, University of Notre Dame, Notre Dame, IN 46556, USA.

**Keywords:** antibiotic resistance, aminoglycosides, acetyltransferase, crystal structure

## Abstract

Aminoglycoside 6’-acetyltransferase-Im (AAC(6’)-Im) is the closest monofunctional homolog of the AAC(6’)-Ie acetyltransferase of the bifunctional enzyme AAC(6’)-Ie/APH(2”)-Ia. The AAC(6’)-Im acetyltransferase confers 4- to 64-fold higher MICs to 4,6-disubstituted aminoglycosides and the 4,5-disubstituted aminoglycoside neomycin than AAC(6’)-Ie, yet unlike AAC(6’)-Ie, the AAC(6’)-Im enzyme does not confer resistance to the atypical aminoglycoside fortimicin. The structure of the kanamycin A complex of AAC(6’)-Im shows that the substrate binds in a shallow positively-charged pocket, with the N6’ amino group positioned appropriately for an efficient nucleophilic attack on an acetyl-CoA cofactor. The AAC(6’)-Ie enzyme binds kanamycin A in a sufficiently different manner to position the N6’ group less efficiently, thereby reducing the activity of this enzyme towards the 4,6-disubstituted aminoglycosides. Conversely, docking studies with fortimicin in both acetyltransferases suggest that the atypical aminoglycoside might bind less productively in AAC(6’)-Im, thus explaining the lack of resistance to this molecule.

## INTRODUCTION

Aminoglycosides are potent, broad-spectrum bactericidal antibiotics used to treat many serious bacterial infections 1. Among the three major structural groups of aminoglycosides, 4,6-disubstituted, 4,5-disubstituted, and atypical, the 4,6-disubstituted compounds are the most commonly used in the clinic (Figure S1). Aminoglycosides bind to the 30S ribosomal subunit, resulting in mistranslation and ultimately, bacterial death 2,3,4. The spread of bacterial strains resistant to aminoglycosides constitutes the major impediment for clinical use of this class of antibiotics. The major mechanism of aminoglycoside resistance in Gram-positive pathogens is the production of aminoglycoside-modifying enzymes, which include aminoglycoside acetyltransferases (AACs), aminoglycoside phosphotransferases (APHs), and aminoglycoside nucleotidyltransferases (ANTs) 5,6,7,8. In Gram-negative bacteria, methylation of the ribosomal RNA provides an additional line of defense against aminoglycoside antibiotics 9.

**Figure 1:**
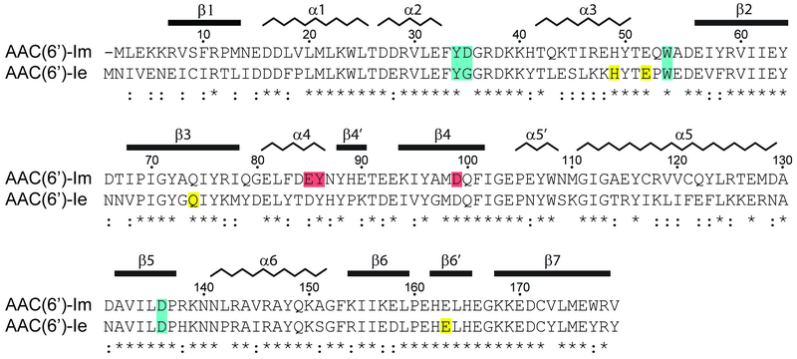
FIGURE 1: Sequence alignment of AAC(6’)-Im and AAC(6’)-Ie. The secondary structure assignment for AAC(6’)-Im is shown above, and the identity (*) and similarity (:) is indicated below each block of sequence. Amino acids involved in kanamycin A binding are shaded blue for those which are common to both enzymes, red for amino acids which interact with kanamycin A only in AAC(6’)-Im, and yellow for amino acids which interact with kanamycin A only in AAC(6’)-Ie 12. The AAC(6’)-Im enzyme is one residue shorter than AAC(6’)-Ie, so for the sake of simplicity, the AAC(6’)-Im sequence numbering begins at Met2 so that the sequence numbering is the same for both enzymes.

The most important aminoglycoside-modifying enzyme in staphylococci and enterococci is the bifunctional AAC(6’)-Ie/APH(2")-Ia, due both to its wide dissemination in these pathogens, and to the wide spectrum of resistance which it confers. It is currently unclear as to what might be the advantage of having two different resistance elements on the same polypeptide in a bifunctional enzyme, as opposed to the expression of two independent monofunctional enzymes 10. Possibilities include the coexpression of two disparate resistance activities to counteract the presence of a single antibiotic, or substrate channeling 11, although there is minimal evidence for the latter. The recent small-angle X-ray scattering (SAXS) study on the bifunctional AAC(6’)-Ie/APH(2")-Ia enzyme suggests that the two active sites are on opposite sides of the enzyme which would preclude any channeling or cooperativity 12. Although the bifunctional enzyme has been identified exclusively in Gram-positive bacteria to date, we recently characterized the APH(2")-If phosphotransferase, the monofunctional counterpart of the phosphotransferase domain of AAC(6’)-Ie/APH(2")-Ia, which was found in the Gram-negative pathogen *Campylobacter jejuni* 13. The monofunctional counterpart (AAC(6’)-Im) of the acetyltransferase domain of the bifunctional enzyme has been identified in both Gram-negative and Gram-positive clinical isolates 14. It shares 60% sequence identity and 83% similarity with AAC(6’)-Ie (Figure 1).

The AAC(6’)-I enzymes can be divided into three sub-families, based upon their structural and kinetic properties 15,16. The AAC(6’)-Ie and AAC(6’)-Ib enzymes are classified together in sub-family C, whose members are all monomeric enzymes. The AAC(6’)-Ig, AAC(6’)-Ih and AAC(6’)-Iy enzymes are in sub-family A and are domain-swapped dimers, with the C-terminal β-strand from one protein chain inserted between two strands from the neighboring molecule. The close proximity of the two protein chains in the dimer brings each monomer close enough such that a loop from one monomer forms part of the other chain’s active site, and vice versa 16. The AAC(6’)-Ii enzyme is currently the sole member of sub-family B, and although this enzyme is also dimeric, the oligomer formed is different from the subfamily A dimer. Moreover the AAC(6’)-Ii enzyme acetylates only aminoglycosides, similar to that of the monomeric sub-family C enzymes, whereas the sub-family A enzymes have been shown to acetylate both aminoglycoside and non-aminoglycoside substrates 16.

The high resolution crystal structure of the kanamycin A complex of AAC(6’)-Ie has been reported 12. Given the strong familial relationship between AAC(6’)-Im and AAC(6’)-Ie, we have undertaken the enzymological and structural analyses of these enzymes. Here we report the antibiotic susceptibility and kinetic profile and the X-ray structure of the AAC(6’)-Im acetyltransferase in its apo form and as the binary kanamycin A complex, and classify the enzyme as a member of the AAC(6’)-I sub-family C. An analysis of the structural features involved in substrate binding provides insight into the modulation of the activity of these enzymes.

## RESULTS AND DISCUSSION

### Antibiotic resistance profile 

When expressed in the *Escherichia coli* JM83 strain, AAC(6’)-Im produces resistance to a wide range of clinically important 4,6-disubstituted aminoglycosides with minimal inhibitory concentration (MIC) values 8 - 128-fold above those for the recipient strain (Table 1). The enzyme increases MICs of the 4,5-disubstituted antibiotic neomycin 8-fold, while the MICs of paromomycin and lividomycin remain at background level. No significant change in MIC values is observed for the atypical aminoglycosides hygromycin (2-fold) and fortimicin (no change). When compared to its closely-related counterpart, AAC(6’)-Ie, from the bifunctional AAC(6’)-Ie/APH(2")-Ia enzyme, the monofunctional AAC(6’)-Im acetyltransferase overall produces significantly higher (4 - 64-fold) levels of resistance to the 4,6-disubstituted aminoglycosides (Table 1). In stark contrast, while AAC(6’)-Im fails to elevate resistance to the atypical aminoglycoside, fortimicin, AAC(6’)-Ie produces a significant (32-fold) increase in the MIC of this antibiotic.

**Table 1:** MIC profile of aminoglycosides for *E. coli* JM83 expressing AAC(6’)-Im and AAC(6’)-Ie. *^a^* These aminoglycosides have a hydroxyl at the 6’ position and thus are not substrates for the AAC(6’) enzymes.

	**MIC (µg/ml)**		
**Aminoglycoside**	**AAC(6’)-Im**	**AAC(6’)-Ie**	***E. coli* JM83**
**4,6-Disubstituted**			
Tobramycin	64	4	0.5
Amikacin	64	4	0.5
Kanamycin A	256	64	4
Kanamycin B	128	16	1
Isepamicin	32	4	2
Dibekacin	64	2	0.5
Netilmicin	32	0.5	0.5
Sisomicin	16	1	0.5
Arbekacin	4	1	0.5
**4,5-Disubstituted**			
Neomycin	4	1	0.5
Paromomycin *^a^*	8	8	8
Lividomycin *^a^*	8	8	8
**Atypical**			
Hygromycin	64	64	32
Fortimicin	2	64	2

### Kinetic studies

To evaluate whether differences in MICs produced by the AAC(6’)-Im and AAC(6’)-Ie acetyltransferases result from differences in the turnover rates or apparent affinity of substrates for these enzymes, we attempted to determine the steady-state kinetic parameters *k*_cat_ and *K*_m_ for AAC(6’)-Im and compare them to those previously reported for AAC(6’)-Ie 17. However, we observed strong substrate inhibition with all 4,6-disubstituted aminoglycosides at concentrations required to determine *k*_cat_ and *K*_m_ values, which prevents their determination. It should be noted that substrate inhibition was also observed for many aminoglycosides with AAC(6’)-Ie 17, however it was less significant, allowing for the determination of these parameters.

**Figure 2:**
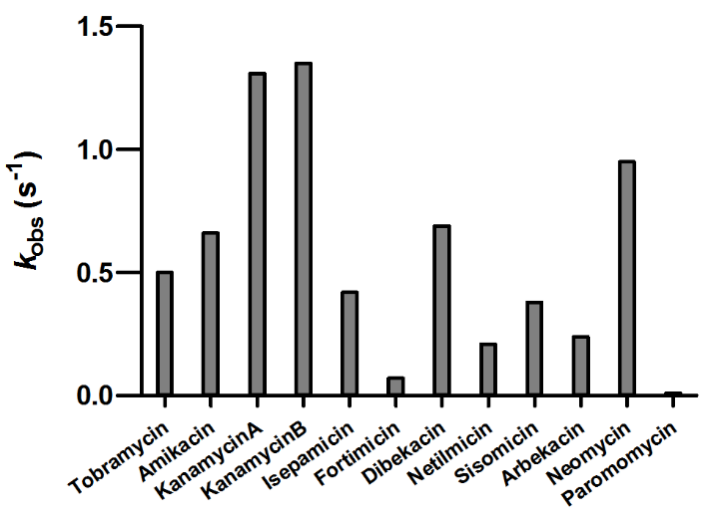
FIGURE 2: The rate constants for the acetyltransferase activity of AAC(6’)-Im for selected aminoglycosides.

As we were unable to evaluate *k*_cat,_ and *K*_m_ values for the majority of aminoglycosides used in our study, we chose to measure the rate constant for acetylation at a single concentration of each substrate (Figure 2). This was done at 5 µM of aminoglycoside to minimize the effect of substrate inhibition. For most aminoglycosides we observed relatively good correlation between the acetylation rate constants and the MIC values (Figure 2 and Table 1). The exception to this was neomycin for which the low resistance level conferred (4 µg/ml) does not match the relatively high acetylation rate (0.95 s^-1^). For neomycin, it has been reported that the acetylated antibiotic still retains significant antimicrobial activity 18,19,20,21, which may explain the low MIC observed for this antibiotic with AAC(6’)-Im.

### Kanamycin A binding by AAC(6’)-Im

**Figure 3:**
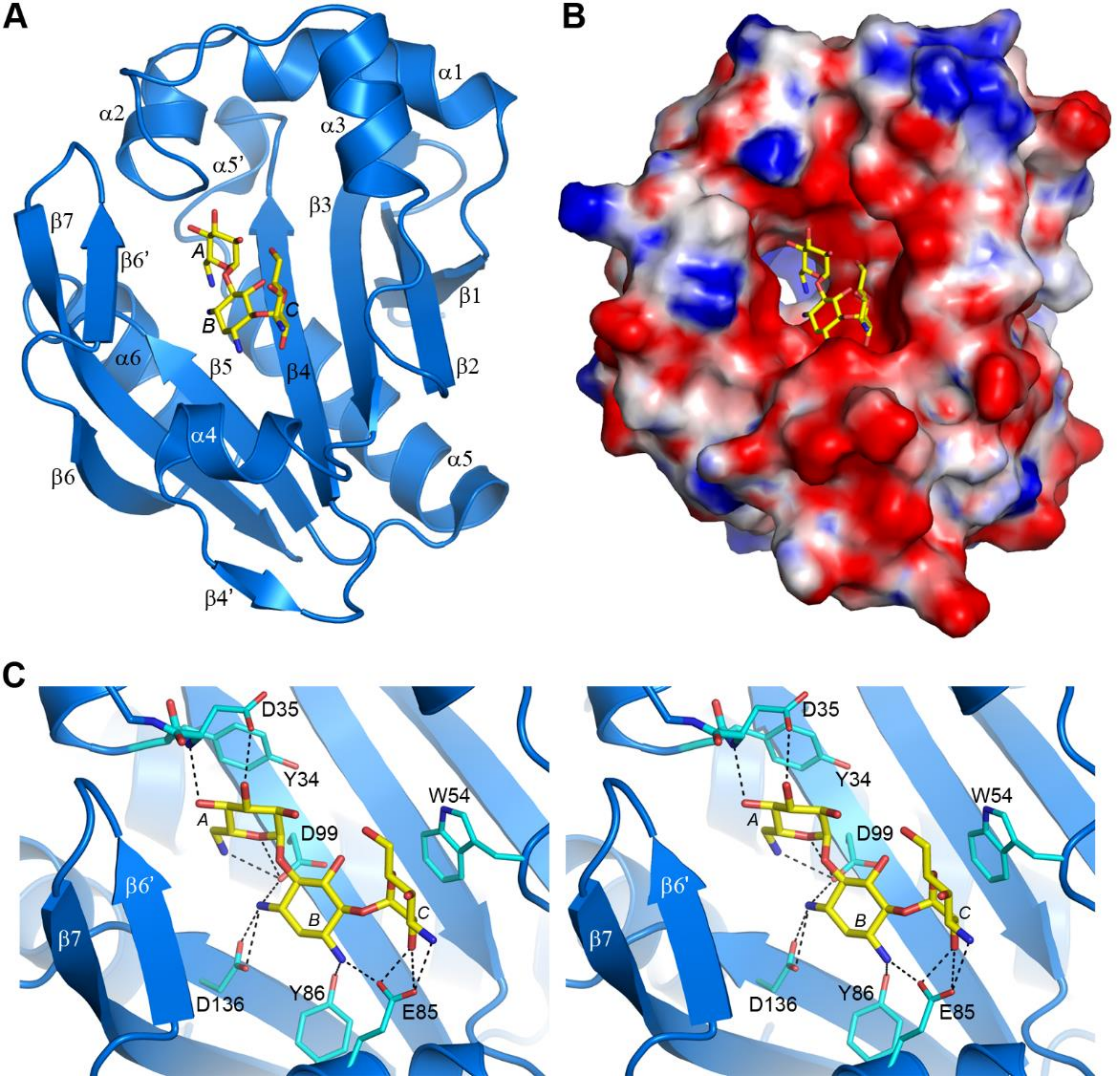
FIGURE 3: The kanamycin A complex of AAC(6’)-Im. **(A) **Ribbon representation of AAC(6’)-Im (blue) with the bound kanamycin A shown as yellow sticks. The secondary structure nomenclature used throughout the paper is indicated. **(B)** Electrostatic surface representation of AAC(6’)-Im, with the surface potentials ranging from -5 kT/e (red) to +5 kT/e (blue). The bound kanamycin A is shown as yellow sticks. **(C)** Stereoview of the kanamycin A binding site of AAC(6’)-Im, showing the hydrogen bonding interactions (black dashed lines) of kanamycin A (yellow sticks) with the protein side chains (cyan sticks).

The AAC(6’)-Im structure was solved in both the apo-form at 1.7 Å resolution, and as the binary complex with kanamycin A (AAC(6’)-Im-kanamycin A) to 1.95 Å resolution (Table S1). The overall structure is shown in Figure 3A. The kanamycin binding site is in a shallow highly negatively-charged pocket in the molecular surface (Figure 3B). Strands β3, β4 and β5 form the base of the pocket, with helices α2, α3 and α4, strand β6’, and the loop between helix α3 and strand β2 forming the walls (Figure 3A). The kanamycin A molecule is anchored by twelve hydrogen bonds, four to the A ring, five to the central B ring, and three to the C ring (Figure 3C and Table S2). The A ring is further stabilized by a hydrophobic face-to-face packing interaction with the side chain of Tyr34 (Figure 3C) which serves to help orient the ring such that the N6’ side group is positioned correctly for acetylation. At the opposite end of the substrate, the C ring is also held in position by a face-to-face hydrophobic interaction with the side chain of Trp54 from the α3-β2 loop (Figure 3C). Superposition of the apo-AAC(6’)-Im and AAC(6’)-Im-kanamycin A structures gives an root-mean-square deviation (*rmsd)* of 0.6 Å. Inspection of the superimposed structures shows that although there are no major differences in the overall structure of the enzyme upon substrate binding, there are some minor rearrangements of the residues inside the kanamycin binding site. A β-hairpin formed by strand β6’ and the N-terminal end of strand β7 moves inward as a rigid body (Figure S2). Although there is no direct contact between residues in this strand-loop-strand motif and kanamycin, there is a water-mediated contact with His165 and the O4’ atom of the substrate (Figure S2). In addition, small inward or outward movements of side chains which surround the kanamycin molecule are also observed (Figure S2), primarily to facilitate efficient hydrogen bonding and hydrophobic interactions with the substrate.

Superposition of AAC(6’)-Im onto the six other AAC(6’) enzymes whose structures have been reported gave *rmsd*s of 1.0 Å, 1.7 Å, 2.4 Å, 2.7 Å, 3.0 Å, and 2.9 Å for AAC(6’)-Ie 12, AAC(6’)-Ib 22,23, AAC(6’)-Ii 24, AAC(6’)-Ig 16, AAC(6’)-Ih 16, and AAC(6’)-Iy 25, respectively (Table S3). The variation in the *rmsd*s is consistent with the familial relationships between these enzymes. A number of these enzyme structures were solved as complexes with aminoglycoside substrates; the coenzyme-A (CoA) and kanamycin A complex of AAC(6’)-Ib (AAC(6’)-Ib-CoA-kanamycin A; PDB code 2QIR), the AAC(6’)-Ib-acetyl-CoA-kanamycin C complex (PDB code 1V0C), the AAC(6’)-Ib-CoA-ribostamycin complex (PDB code 2BUE), the AAC(6’)-Ib-acetyl-CoA-paromomycin complex (PDB code 2VQY), the AAC(6’)-Ie-CoA-kanamycin A complex (PDB code 4QC6), the AAC(6’)-Ig-tobramycin complex (PDB code 4EVY), and the AAC(6’)-Iy-CoA-ribostamycin complex (PDB code 1S3Z).

### Structural comparison of AAC(6’)-Im with AAC(6’)-Ie

To gain some structural insights into the differences in MICs between the closely related enzymes AAC(6’)-Im and AAC(6’)-Ie from sub-family C, the kanamycin A complexes of these enzymes were analyzed in detail. In AAC(6’)-Ie, the kanamycin A substrate is anchored by ten hydrogen bonds to the protein (four with the A ring, one with the central B ring and five with the C ring), along with a number of water molecules (Figure S3) 12. The hydrogen bonding network differs substantially from that described for AAC(6’)-Im (Figure 3C and Table S2), in particular at the B ring which in AAC(6’)-Ie is anchored by a single hydrogen bond compared to five in AAC(6’)-Im. Although the number of hydrogen bonds with the A ring is similar in both enzymes, the residues involved differ. These changes in the hydrogen bonding patterns could contribute to the differences in the observed MICs for the two enzymes.

**Figure 4:**
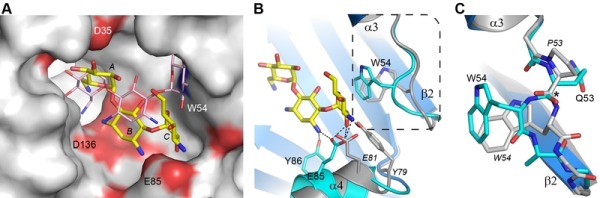
FIGURE 4: Structural comparison of AAC(6’)-Im and AAC(6’)-Ie. **(A) **Molecular surface representation of the binding site of AAC(6’)-Im (grey, red and blue shading). The kanamycin A molecule in AAC(6’)-Im binding site is shown as yellow sticks and the kanamycin A molecule in AAC(6’)-Ie is shown as thin pink sticks. **(B)** Ribbon representation of the loop between helix α3 and strand β2 shown in cyan for AAC(6’)-Im and grey for AAC(6’)-Ie. The location of the Trp54 side chain is indicated for both enzymes. The kanamycin A molecule in AAC(6’)-Im is shown as yellow sticks. The dashed box shows the area represented in panel C. **(C)** Close up view of the conformational difference caused by the presence of Pro53 in AAC(6’)-Ie (grey) relative to a Gln53 in AAC(6’)-Im (cyan). The difference in orientation of the residue 53 carbonyl group is indicated by the asterisk. Residues from AAC(6")-Ie are labeled in italics.

Despite the fact that there are only two sequence differences between the two enzymes in the vicinity of the binding site (Asp35 and Glu85 in AAC(6’)-Im are glycine and aspartate, respectively, in AAC(6’)-Ie (Figure 1)), there is a significant shift in the orientation of the substrate in these two enzymes (Figure 4A). When the kanamycin A conformations are compared it can be seen that in AAC(6’)-Im, the A ring (containing the N6’ site of modification) is rotated approximately 45° relative to the A ring position in AAC(6’)-Ie. This, in turn, moves the B and C rings in AAC(6’)-Im towards helix α4, Asp136 and Glu85 (Figure 4A). This movement, which results in a difference of approximately 4.5 Å in the positions of the C rings in the two enzyme complexes, is primarily facilitated by a change in conformation of the loop between helix α3 and strand β2, and structural and sequence differences in the α4 helix (Figure 4B). The presence of a conformationally-restricted proline (Pro53) at the C-terminus of helix α3 in AAC(6’)-Ie leads to an alteration of the main chain dihedral ψ angle at this residue (Figure 4C). Prolines typically have their main chain dihedral angle φ constrained to near -60°, with the ψ angle adopting either -45° or 135°. The Pro53 residue in AAC(6’)-Ie has φ and ψ angles of -75° and 158°, respectively, such that the carbonyl oxygen points into the substrate binding site and the main chain which follows, projects outward (Figure 4C). Consequently, the side chain of Trp54 in AAC(6’)-Ie moves approximately 3 - 4 Å away from the substrate binding site (Figures 4B and 4C) and the C ring of kanamycin A moves to maintain the hydrophobic interaction. Moreover, sequence differences at the N-terminus of helix α4 in AAC(6’)-Ie result in two bulky residues (Tyr79 and Glu81) projecting into the binding site, further preventing the C and B rings from adopting the conformation observed in AAC(6’)-Im (Figures 4A and 4B).

In the AAC(6’)-Im enzyme, the equivalent residue at position 53 on the α3-β2 loop is a glutamine (φ/ψ angles, -98° and 72°), with its carbonyl oxygen pointing outward and the Trp54 side chain projecting inward (Figure 4C). In this orientation it would severely clash with the C ring of kanamycin were the substrate to bind in the same manner as observed in AAC(6’)-Ie (Figure 4A). The C ring, therefore, must adopt a position away from the tryptophan, moving towards helix α4 where it interacts with residue Glu85 at the C-terminal end of the helix (Figures 3C and 4B). Differences in sequence and structure at the N-terminal end of helix α4 allow this part of the binding site to be more open than in AAC(6’)-Ie and readily able to accommodate the movement of the substrate.

### Structural comparison of AAC(6’)-Im with other AAC(6’) enzymes

To perform structural comparison of various aminoglycoside 6’ acetyltransferases, the structure the kanamycin A complex of AAC(6’)-Im was superimposed onto the ribostamycin and paromomycin complexes of AAC(6’)-Ib (Figures S4A and S4B, respectively), the tobramycin complex of AAC(6’)-Ig (Figure S4C) and the ribostamycin complex of AAC(6’)-Iy (Figure S4D). It should be noted that paromomycin has an oxygen atom at the 6’ position on the A ring (Figure S1), and thus is not a substrate for these enzymes. There are significant differences in the structure of the substrate binding site in AAC(6’)-Ib (relative to AAC(6’)-Im), and these are highlighted in Figure S4A. The four main regions of structural variation are: (i) the loop between helix α3 and strand β2 in AAC(6’)-Im is replaced by an α-helix in AAC(6’)-Ib; (ii) the α2-α3 loop is lengthened by a turn of 3_10_ helix; (iii) the absence of the strand equivalent to β6’ and the shortening of strand β7; and (iv) the replacement of helix α4 and strand β4’ by an unstructured loop which projects into the binding site. However, despite these changes, the A and B rings of the 4,5-disubstituted aminoglycosides ribostamycin (Figure S4A) and paromomycin (Figure S4B) in AAC(6’)-Ib overlap almost exactly with the equivalent rings of kanamycin A in AAC(6’)-Im. In both cases, the ribose moiety attached at the 5 position deviates markedly, projecting away from the kanamycin A substrate C ring position observed in AAC(6’)-Im, towards the C-terminus of helix α3.

In the more distantly-related domain-swapped dimeric enzymes from sub-family A of the AAC(6’)-I enzymes, AAC(6’)-Ig and AAC(6’)-Iy, the binding sites differ substantially. These two enzymes share 40% sequence identity with each other and are structurally very similar (Table S3), yet only show between 10 - 16% identity with AAC(6’)-Im, AAC(6’)-Ie and AAC(6’)-Ib. In both AAC(6’)-Ig and AAC(6’)-Iy, the β6’-β7 hairpin is absent and the helix equivalent to helix α3 in AAC(6’)-Im is one turn longer. This positions the loop, which is topologically equivalent to the α3-β2 loop in AAC(6’)-Im, approximately 4 Å further from the binding pocket (Figures S4C and S4D). Similar to AAC(6’)-Ib, the equivalent of helix α4 is also missing from both AAC(6’)-Ig and AAC(6’)-Iy, and is replaced by an extended loop which in these enzymes has moved approximately 3 - 4 Å away from the binding site. Moreover, a loop from the adjacent protein chain of the dimer inserts into the active site, and residues from this loop interact with the tobramycin and ribostamycin in these complexes. These combined structural differences make the substrate binding site in the AAC(6’)-Ig and AAC(6’)-Iy significantly larger 16 and consequently, the tobramycin and ribostamycin in these two enzymes bind in a completely different manner.

### Acetylation of aminoglycosides by AAC(6’) enzymes

**Figure 5:**
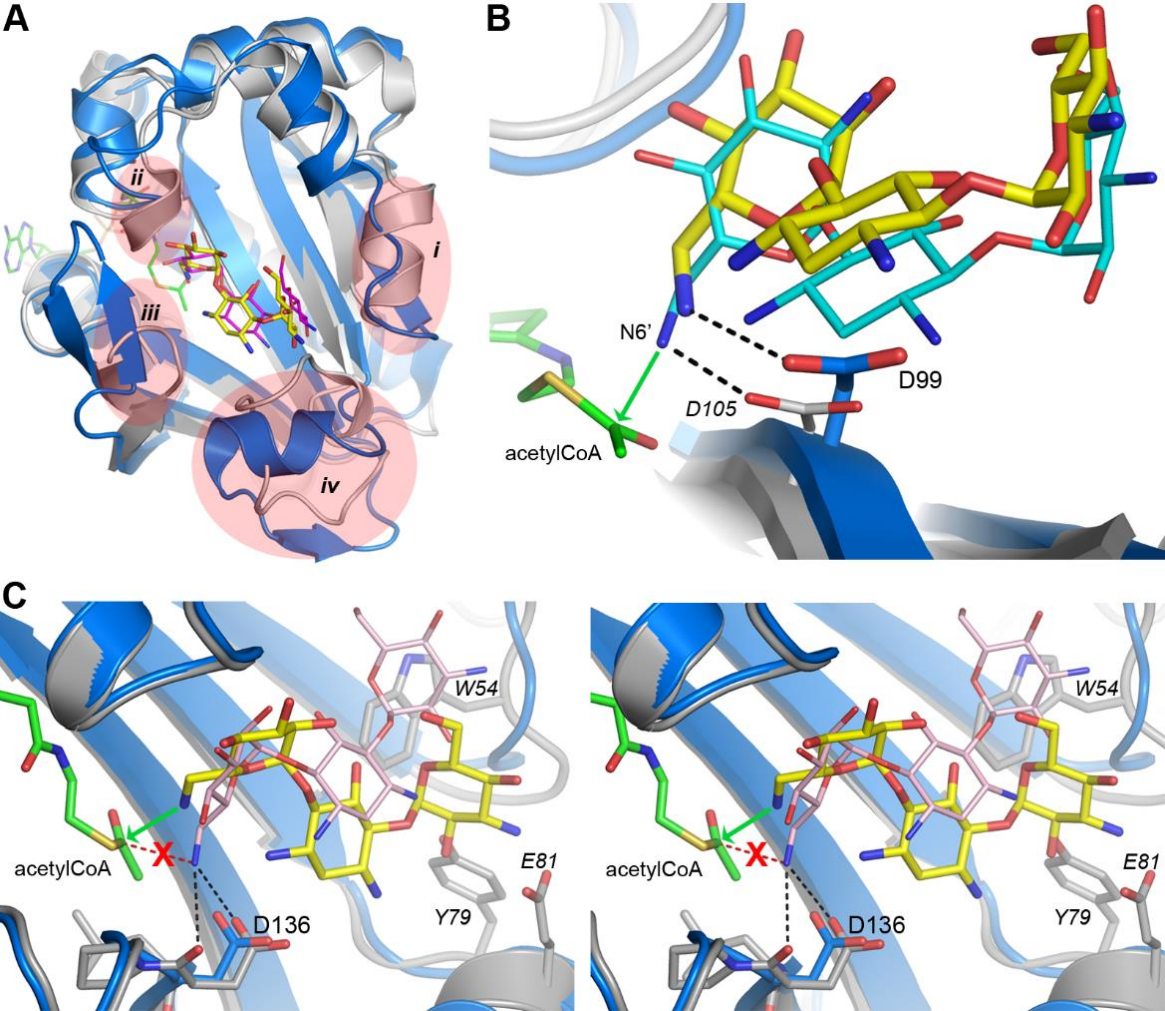
FIGURE 5: Structural comparison of AAC(6’)-Im and AAC(6’)-Ib. **(A) **Superposition of AAC(6’)-Im (blue) and AAC(6’)-Ib (grey). Kanamycin A is shown in yellow sticks for AAC(6’)-Im and kanamycin C in magenta sticks for AAC(6’)-Ib. The acetyl-CoA cofactor present in AAC(6’)-Ib is shown as green sticks. The four main regions of structural difference near the substrate binding site are indicated in pale red, as described in Figure S4A. **(B)** Close up view of superimposed kanamycin A substrates in AAC(6’)-Im (yellow with the enzyme shown in blue) and AAC(6’)-Ib (cyan with the enzyme shown in grey). In AAC(6’)-Ib the N6’ group is positioned by a hydrogen bond to the Asp105 side chain (labeled in italics), and the favorable close approach of the N6’ to the acetyl carbon is shown by the green arrow. A similar hydrogen bonding interaction is seen in AAC(6’)-Im with the Asp99 side chain. **(C)** Stereoview close up of the active site in AAC(6’)-Ie (grey) superimposed onto AAC(6’)-Im (blue). The kanamycin A substrates in AAC(6’)-Im (yellow) and AAC(6’)-Ie (pink) are shown. The difference in orientation of the two aminoglycosides directs the N6’ group in AAC(6’)-Ie away from the acetyl-CoA, where it is anchored by two hydrogen bonds to Asp136. The favorable approach of the N6’ group to the acetyl carbon in the AAC(6’)-Im-bound kanamycin A is indicated by the green arrow, and what would be a unfavored orientation in AAC(6’)-Ie is indicated by a red dashed line. Residues from AAC(6")-Ie are labeled in italics.

The AAC(6’)-Im-kanamycin A complex was superimposed onto the kanamycin A and kanamycin C complexes of AAC(6’)-Ib (PDB codes 2QIR and 1V0C, respectively). The antibiotic molecules occupy essentially the same position and have the same relative conformation in these enzymes (Figure 5A), despite the fact that there are some significant structural differences in the AAC(6’)-Ib substrate binding site (Figures S4A and 5A). The crystal structure of AAC(6’)-Ib with acetyl-CoA and kanamycin C (PDB code 1V0C) provides valuable insight into the mechanism of acetylation in these enzymes. Although kanamycin C is a variant of the antibiotic that has a hydroxyl group at the 6’ position and is therefore not a substrate for the AAC(6’) enzymes, it binds to AAC(6’)-Ib in the presence of acetyl-CoA to give rise to an abortive ternary complex. In this structure, the O6’ atom is approximately 2.2 Å from the carbon atom of the acetyl moiety, positioned to make an efficient nucleophilic attack were it an amide group 22. After superposition, the kanamycin A substrate in the AAC(6’)-Ib-CoA-kanamycin A complex (PDB code 2QIR) is in almost exactly the same place as the kanamycin C molecule, such that the N6’ atom would be approximately 2.0 Å from the acetyl carbon, poised perfectly for attack (the position of the acetyl-CoA from the abortive AAC(6’)-Ib-acetyl-CoA-kanamycin C complex was also used for this comparison).

The position of the acetyl-CoA in the context of the AAC(6’)-Im structure is currently unknown, therefore the acetyl-CoA from the abortive AAC(6’)-Ib-acetyl-CoA-kanamycin C complex was used to represent the approximate location of the cofactor in AAC(6’)-Im, giving rise to a composite model of an AAC(6’)-Im-acetyl-CoA-kanamycin A complex. In this composite model, the distance between the N6’ group of the kanamycin A in AAC(6’)-Im and the acetyl carbon is approximately 2.8 Å. A hydrogen bond between the N6’ atom and the side chain of a conserved aspartate (Asp99 in AAC(6’)-Im (Figure 3C) and Asp105 in AAC(6’)-Ib) serves to orient the nucleophilic nitrogen atom so that it would be correctly positioned for direct transfer of the acetyl group to the kanamycin molecule (Figure 5B) in both of these enzymes.

When the kanamycin A complex of AAC(6’)-Ie is superimposed onto AAC(6’)-Im, the rotation of the A ring (described above) places the N6’ atom over 2 Å from the equivalent position in AAC(6’)-Im, anchored by two hydrogen bonds to the carbonyl oxygen and the side chain of Asp136 (Figures 5C and S3). More importantly, the nitrogen atom is directed away from the acetyl-CoA, approximately 3.1 Å from the acetyl carbon, and in an orientation which would not favor an efficient interaction with the cofactor. The hydrogen bonding interaction with the Asp99 side chain, which serves to correctly orient the N6’ group in AAC(6’)-Im, is missing in AAC(6’)-Ie. This difference in orientation of kanamycin in AAC(6’)-Ie could explain the almost universally lower MIC’s for the majority of the 4,6-disubstituted aminoglycosides. Since the 4,6-disubstituted aminoglycosides all have essentially similar structures, it seems highly likely that the relocation of the Trp54 side chain and the insertion of the bulky residues Tyr79 and Glu81 at the N-terminus of helix α4 in AAC(6’)-Ie (Figure 4B) could facilitate a rotation of these substrates similar to that seen for kanamycin. In order for the N6’ atom of these 4,6-disubstituted aminoglycosides to be positioned for effective nucleophilic attack on the acetyl carbon, the hydrogen bonds holding the N6’ atom in the unproductive orientation would need to be broken so that the C5’-C6’ bond could rotate and allow the nitrogen to approach the acetyl carbon. This energy requirement could therefore give rise to the apparently lower activity of the AAC(6’)-Ie enzyme.

These structural analyses of the kanamycin A complexes of AAC(6’)-Im and AAC(6’)-Ie show that conformational rearrangements within the substrate binding sites of these enzymes play key roles in the orientation of the bound kanamycin molecule. These structural changes alter the way in which the 4,6-disubstituted aminoglycosides are positioned in the binding site, affecting the orientation of the N6’ amino group relative to the acetyl donor, which could be responsible for differences in the activity of the enzymes. Given the high degree of sequence identity between the AAC(6’)-Im and AAC(6’)-Ie acetyltransferases, an evolutionary relationship between these two enzymes is highly likely, with both enzymes probably having diverged from a common ancestral precursor. The significantly lower MICs towards the 4,6-disubstituted aminoglycosides observed for the AAC(6’)-Ie domain of the bifunctional enzyme (compared to the monofunctional AAC(6’)-Im) suggest that any evolutionary pressure, which might typically be expected to lead to an increase in enzyme efficiency, has been somewhat relieved by the presence of APH(2")-Ia, which is a highly efficient enzyme capable by itself of protecting bacteria against the 4,6-disubstituted antibiotics.

### Fortimicin binding to AAC(6’)-Im and AAC(6’)-Ie

The AAC(6’)-Im acetyltransferase shows elevated MICs for almost all aminoglycoside substrates compared to AAC(6’)-Ie, except in the case of the atypical substrate fortimicin (Table 1). Contrary to what is observed with 4,6-disubstituted aminoglycosides, AAC(6’)-Ie elevates resistance to this antibiotic 32-fold above the background level. Although fortimicin comprises only two rings, a decorated aminocyclitol and a single glycan group, it does possess an amino group at the 6’ position which would be the potential site of acetylation by the AAC(6’) enzymes (Figure S1). Since no structural information is available for this aminoglycoside bound to any AAC(6’) enzyme, we undertook ligand docking studies using the program ICM-Pro. Both kanamycin and fortimicin were docked to receptor models derived from AAC(6’)-Im, AAC(6’)-Ib and AAC(6’)-Ie. Kanamycin A was used as a test of the docking procedures. This substrate was docked 15 independent times to the three models and in the majority of docking runs (> 10/15), poses identical or substantially similar to the known crystal structures were obtained (data not shown).

When fortimicin was docked in 15 independent runs to the AAC(6’)-Ie model, a significant number (7/15) of self-consistent poses which placed the N6’ amino group within 0.5 Å of the N6’ atom of the crystal structure of the bound kanamycin were observed. In all seven poses, the N6’ atom was within 3 Å of the acetyl carbon of the modeled acetyl-CoA, and in an orientation relative to the acetyl carbon which could lead to efficient acetyl transfer. Conversely, when fortimicin was docked to AAC(6’)-Im in 15 independent runs, all of the poses obtained were randomly distributed throughout the binding site, and none of the poses were self-consistent (data not shown). Moreover, none of the poses appeared to be productive, in that there were no cases where the fortimicin N6’ atom came close enough to the acetyl carbon of the modeled acetyl-CoA, or in the correct orientation, to facilitate efficient acetylation of the amino group. These docking results are entirely consistent with the differences in the MICs observed for AAC(6’)-Im and AAC(6’)-Ie. Given that a large number of clearly viable poses were observed for AAC(6’)-Ie, these docking results could explain the differences in resistance to fortimicin produced by these related acetyltransferases.

## MATERIALS AND METHODS

### Enzyme cloning and antibiotic susceptibility profile analysis

The genes for the AAC(6’)-Im and AAC(6’)-Ie were cloned into the vector pBluescript II KS(+) under the same promoter to minimize potential differences in enzyme expression levels. For AAC(6’)-Ie we generated two constructs by cloning the gene encoding either the first 197 or 179 amino acids of the bifunctional enzyme. Both constructs produced similar MICs when expressed in *Escherichia coli* JM83 and the construct encoding the 179 amino acid enzyme was used for MIC testing and protein purification. The MICs of various aminoglycoside antibiotics were determined by the broth microdilution technique according to the guidelines of the Clinical and Laboratory Standards Institute 26, with *E. coli* JM83 without vector as a control (Table 1).

### Protein purification and enzyme kinetics 

For large scale protein purification the gene for AAC(6’)-Im was optimized for expression in *E. coli*, cloned into the pET22b(+) expression vector, and the protein was purified as previously described 27. The AAC(6’)-Im acetyltransferase activity towards aminoglycosides was monitored spectrophotometrically using a coupled assay 28. In this assay acetyl-CoA serves as the source of the acetyl group. Upon acetylation, released coenzyme A reacts with 4,4’-dithiodipyridine releasing 4-thiopyridone (ϵ_324_ = +19800 M^-1^ cm^-1^), which can be monitored at 324 nm. Assay mixtures contained 100 mM HEPES (pH 7.0), 2 mM 4,4’-dithiodipyridine, variable concentrations of aminoglycoside (1 - 100 µM), 100 µM acetyl-CoA, and 2.5 - 40 mM MgCl_2_ in a total volume of 250 µl. Reactions were initiated by the addition of the enzyme and followed at 25°C. The enzyme’s acetyltransferase activity for the respective aminoglycosides was measured from the initial rate of each reaction.

### Crystallization, data collection, structure solution and refinement

AAC(6’)-Im was crystallized as previously described 27. Three different crystal forms were initially obtained, and data collection from form III (an AAC(6’)-Im-kanamycin complex in space group P6_5_) has been described 27. This data was reprocessed to 1.95 Å resolution using XDS 29 and AIMLESS 30 and data are presented in Table S1. A data set to 1.7 Å resolution was collected from a form II apo-AAC(6’)-Im crystal at SSRL beamline BL12-2 using X-rays at 12658 eV (0.9795 Å). A total of 950 fine phi-sliced (0.2º rotation) images with a 0.2 sec exposure were measured using a Pilatus 6M PAD detector running in shutterless mode. The images were processed using XDS 29, and scaled and merged with AIMLESS 30. Data collection statistics are also given in Table S1.

The AAC(6’)-Im-kanamycin A structure was solved as described 27 and refined using PHENIX 31, giving a final model comprising 1507 protein atoms, one kanamycin ligand and 83 water molecules, with final R_work_ and R_free_ values of 17.05% and 19.43% respectively. The apo-AAC(6’)-Im structure was solved by molecular replacement using the refined AAC(6’)-Im-kanamycin A structure, with the kanamycin A and water molecules removed. Two independent molecules were found in the asymmetric unit and subsequently refined using PHENIX 31. The final R_work_ and R_free_ values were 18.46% and 22.93% respectively (see Table S1 for final statistics for both structures). The atomic coordinates and the structure factors for apo AAC(6')-Im and the AAC(6')-Im-kanamycin A complex were deposited to the Protein Data Bank with PDB codes 6BFF and 6BFH, respectively.

### Ligand docking

Ligand docking to AAC(6’)-Im, AAC(6’)-Ib and AAC(6’)-Ie was performed using ICM-Pro 3.8-4 (Molsoft) 32. The AAC(6’)-Ib and AAC(6’)-Ie structures were initially superimposed onto the kanamycin A complex of AAC(6’)-Im. An acetyl-CoA molecule derived from the AAC(6’)-Ib-acetyl-CoA-kanamycin C complex (PDB code 1V0C) was added to the AAC(6’)-Im and AAC(6’)-Ie structures to produce composite models, and all other ligands were removed, along with all water molecules. These models were then loaded into ICM-pro and converted to ICM receptor objects, with optimization of hydrogen atom placement. The approximate location of the aminoglycoside binding site was defined using the kanamycin A position observed in AAC(6’)-Im, and receptor maps were calculated within ICM-Pro. The aminoglycoside substrates kanamycin and fortimicin were subsequently docked to all three AAC(6’) models. The ligand docking runs were performed multiple times, and the binding modes for the three receptor models with the two substrates were extracted from ICM-Pro ad PDB files.

## SUPPLEMENTAL MATERIAL

Click here for supplemental data file.

All supplemental data for this article are also available online at http://microbialcell.com/researcharticles/aminoglycoside-resistance-profile-and-structural-architecture-of-the-aminoglycoside-acetyltransferase-aac6-im/.
