# Supplementary material for: Aminoglycoside resistance profile and structural architecture of the aminoglycoside acetyltransferase AAC(6')-Im
Source: Microb Cell. 2017 Nov 9;4(12):402–10. doi: 10.15698/mic2017.12.602 (PMC5722643; doi:10.15698/mic2017.12.602)
Supplement: Supplementary file 1 [file mic-04-402-s01.pdf]

Supplementary information for

Aminoglycoside resistance profile and structural architecture of the aminoglycoside acetyltransferase AAC(6')-Im

Clyde A. Smith[1,*], Monolekha Bhattacharya[2], Marta Toth[2], Nichole K. Stewart[2], and Sergei B. Vakulenko[2,*]

[1]Stanford Synchrotron Radiation Lightsource, SLAC National Accelerator Laboratory, Menlo Park, CA 94025

[2]Department of Chemistry and Biochemistry, University of Notre Dame, Notre Dame, IN 46556

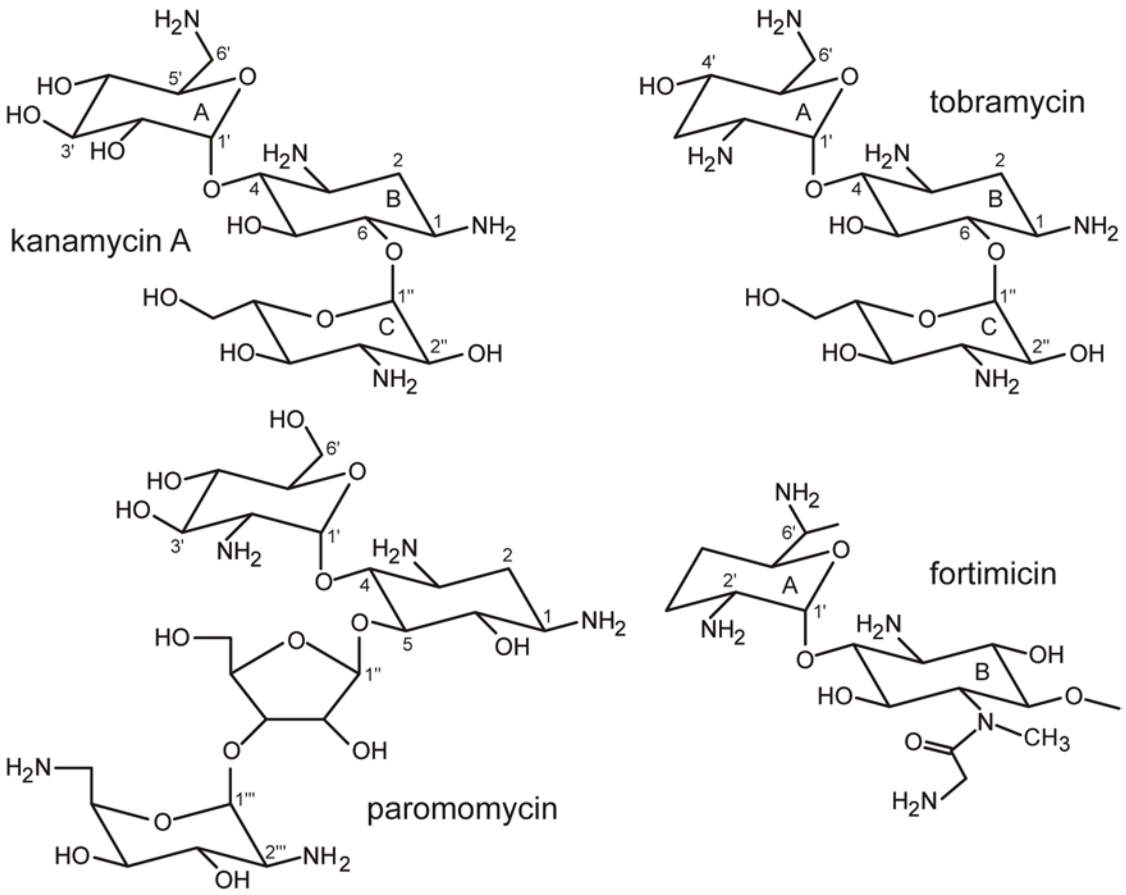

Figure S1. Structures of aminoglycosides. Kanamycin A and tobramycin are examples of 4,6-disubstituted aminoglycoside, paromomycin is a 4,5-disubstituted aminoglycoside, and fortimicin is an example of an atypical aminoglycoside.

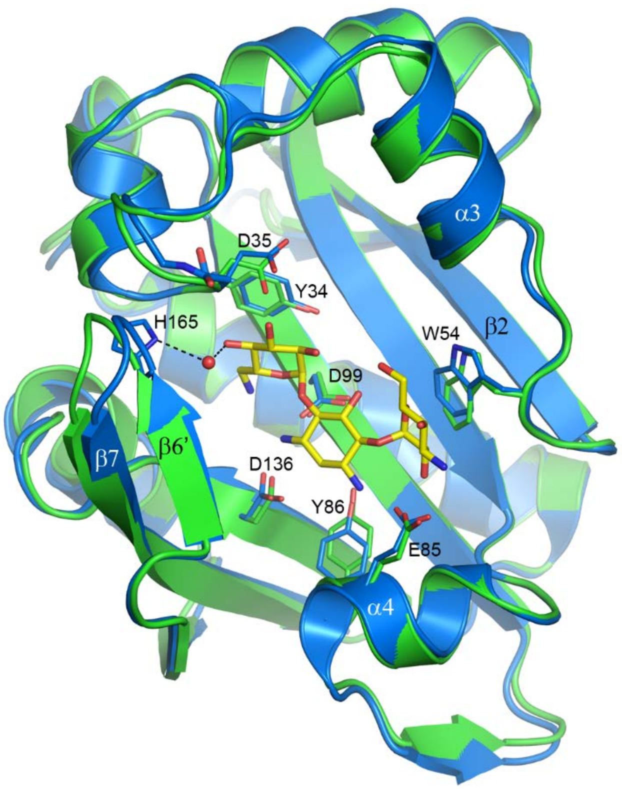

Figure S2. Structure of AAC(6')-Im. Superposition of apo-AAC(6')-Im (green) and the AAC(6')-Im-kanamycin A complex (blue), showing kanamycin A as yellow sticks. The residues involved in kanamycin A binding are also indicated for apo-AAC(6')-Im (green sticks) and AAC(6')-Im-kanamycin A (blue sticks).

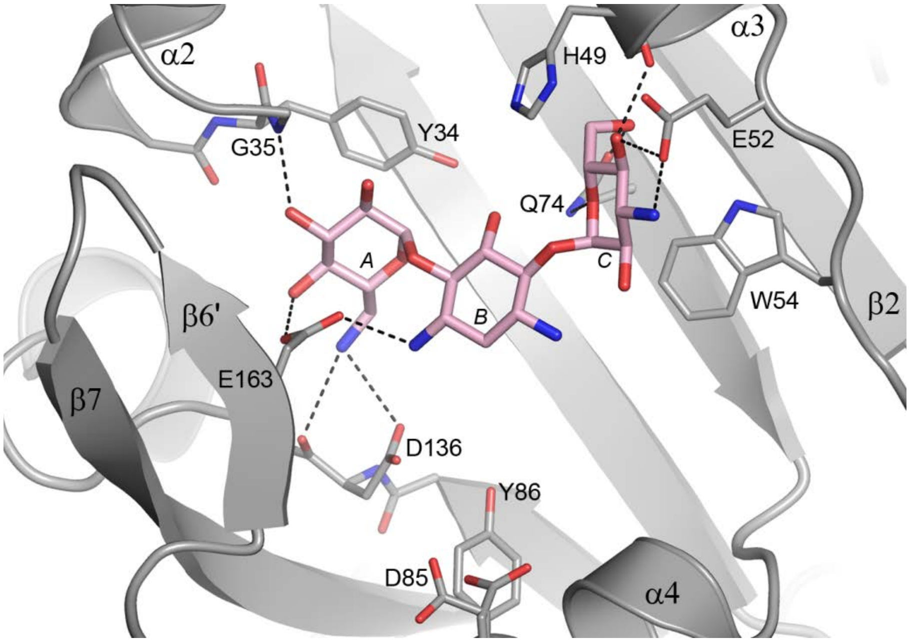

Figure S3. The kanamycin A binding site of AAC(6')-Ie. The AAC(6')-Ie structure is shown as gray ribbons. Hydrogen bonding interactions of kanamycin A (shown as pink sticks) with the protein side chains (gray sticks) are shown as black dashed lines. The A, B and C rings of the kanamycin A are labeled in italics. The orientation is similar to that in Figure 4, which obscures two hydrogen bonds between the side chain of Gln74 and the C ring.

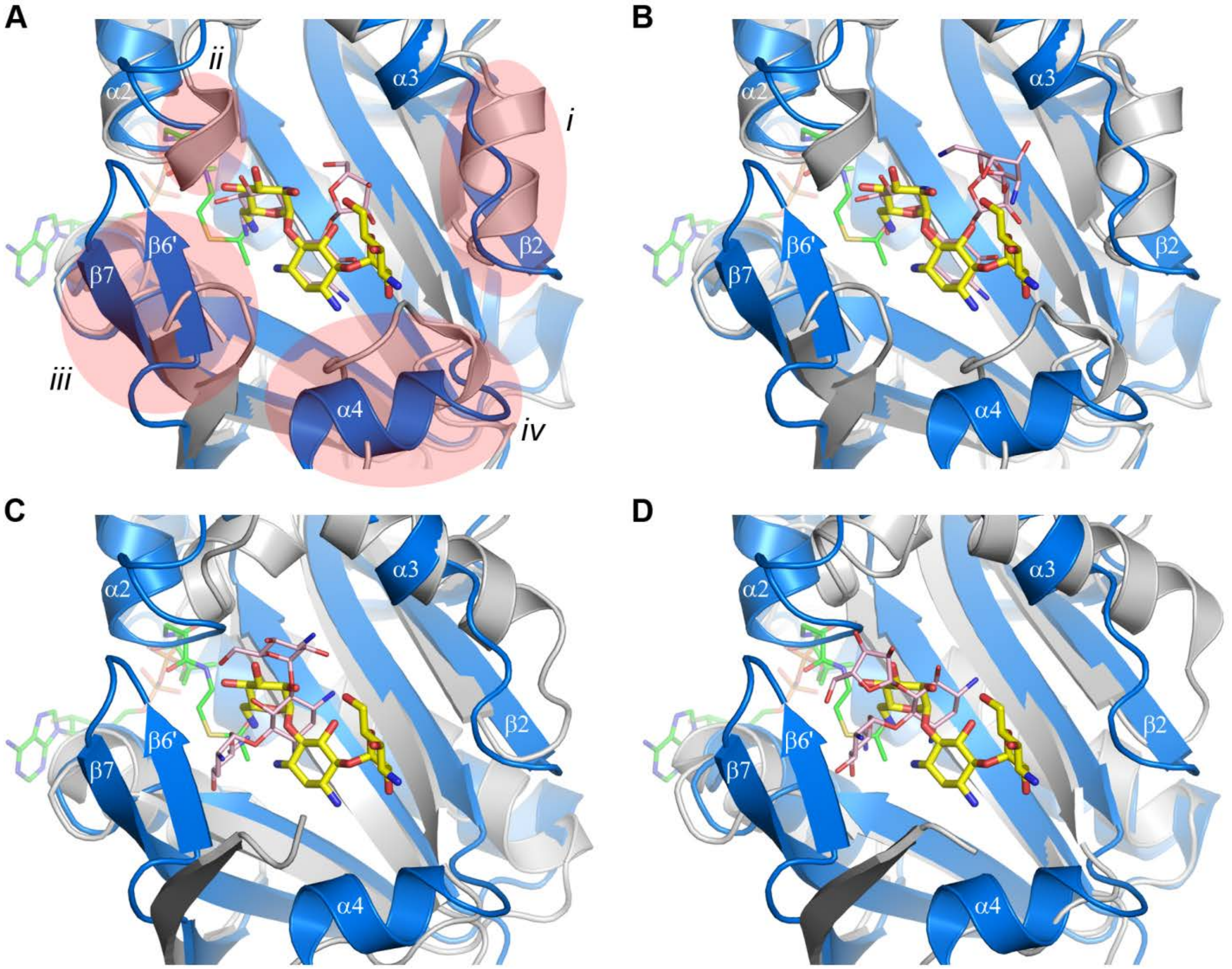

Figure S4. Structural comparison of AAC(6')-Im with other AAC(6') enzymes. The AAC(6')-Im-kanamycin A structure is colored blue in all panels while the other AAC(6') enzymes are colored light gray. The bound kanamycin A is shown as yellow sticks. An acetyl-CoA molecule (green) from the abortive AAC(6')-Ib-acetyl-CoA-kanamycin C complex (PDB code 1V0C) is also shown in all panels. **(A)** Superposition of AAC(6')-Im-kanamycin A with AAC(6')-Ib-ribostamycin. The ribostamycin is shown as thin pink sticks. The four main regions of structural difference near the substrate binding site are indicated in pale red and numbered as follows: (i) the loop between helix α3 and strand β2 in AAC(6')-Im is replaced by an α-helix in AAC(6')-Ib; (ii) the α2-α3 loop is lengthened by a single turn of 3_{10} helix; (iii) the absence of the strand equivalent to β6' and the shortening of strand β7; and (iv) the replacement of helix α4 and

strand β4’ by an unstructured loop which projects into the binding site. **(B)** Superposition of AAC(6’)-Im-kanamycin A with AAC(6’)-Ib-paromomycin. The paromomycin is shown as thin pink sticks. **(C)** Superposition of AAC(6’)-Im with AAC(6’)-Ig-tobramycin. The tobramycin is shown as thin pink sticks. **(D)** Superposition of AAC(6’)-Im-kanamycin A with AAC(6’)-Iy-ribostamycin. The ribostamycin is shown as thin pink sticks.

Table S1. Crystallographic data collection and refinement statistics for AAC(6')-Im

		apo-AAC(6')-Im	AAC(6')-Im-kanamycin A[a]
**Data collection**			
Space Group		C2	$P6_5$
Cell dimensions	a, b, c (Å)	161.52, 34.94, 67.31	107.75, 107.75, 37.33
	α, β, γ (°)	90.0, 102.3, 90.0	90.0, 90.0, 120.0
Resolution (Å)		39.6 – 1.70	19.9 – 1.95
Reflections, observed/unique		141536 / 39815	130956 / 18370
R_{merge} (%)		6.1 (57.7) [b]	6.6 (74.3)
I/σ_I		12.9 (2.3)	18.2 (3.6)
Completeness (%)		96.9 (91.8)	99.8 (98.4)
CC½ [c]		99.8 (78.4)	99.9 (89.6)
Multiplicity		3.6 (3.5)	7.3 (7.1)
Wilson B value		20.6	25.1
**Refinement**			
R_{work} / R_{free} (%)		18.57 / 23.32	17.81 / 20.50
Protein atoms/solvent atoms		3034 / 281	1499 / 83
Average B	– protein (Å^2)	26.5, 28.5	37.2
	– solvent (Å^2)	34.3	41.0
rmsd bonds (Å)		0.008	0.008
rmsd angles (º)		0.903	1.13
Ramachandran plot[d]			
- favoured and allowed regions (%)		98.7	97.5
- number in disallowed regions		2	1

[a] Data collection statistics for form III were previously reported to 2.0 Å resolution [1] and have been reprocessed to 1.95 Å resolution prior to the refinement of the structure.

[b] Values in parentheses are for the outer resolution shells, 1.75 - 1.70 Å for apo-AAC(6')-Im and 2.00 - 1.95 Å for AAC(6')-Im-kanamycin A.

[c] CC½ is the correlation between random half-sets of data [2].

[d] As calculated by MOLPROBITY [3].

Table S2. Hydrogen bonding interactions between kanamycin A and AAC(6')-Im

kanamycin ring	atom[a]	partner	distance (Å)
	O3' (O7)	Asp35 O_{d1}	3.23
A	O4' (O8)	Asp35 N	2.00
	O6' (O5)	Asp99 O_{d1}	3.15
	N6' (N1)	Asp99 O_{d1}	3.04
	N1 (N3)	Glu85 O_{e1}	2.86
		Tyr86 Oη	3.09
B	N3 (N2)	Asp136 $O_{\delta 1}$	3.08
		Asp136 $O_{\delta 2}$	3.13
		Asp99 O_{d1}	2.84
	O2" (O13)	Glu85 O_{e1}	2.55
C		Glu85 O_{e2}	3.22
	N3" (N4)	Glu85 O_{e2}	2.66

[a] The atom name in the PDB file is given in parentheses.

Table S3. Structural and sequence similarities in the AAC(6') enzymes[a]

	Enzyme	-Im	-Ie	-Ib	-Ii	-Ig	-Ih	-Iy
C[b]	-Im	-	1.0 (174)	1.7 (156)	2.4 (131)	2.7 (131)	3.0 (128)	2.9 (134)
	-Ie	60.3%	-	1.9 (157)	2.3 (130)	2.6 (134)	2.8 (129)	2.8 (133)
	-Ib	26.3%	24.8%	-	2.4 (129)	2.5 (126)	2.7 (128)	2.5 (127)
B	-Ii	13.7%	15.4%	17.8%	-	1.8 (122)	1.8 (123)	1.7 (127)
A	-Ig	9.9%	11.2%	14.3%	13.1%	-	0.6 (146)	1.0 (144)
	-Ih	11.7%	11.6%	12.5%	17.9%	71.2%	-	1.3 (146)
	-Iy	13.4%	12.8%	16.5%	17.3%	40.3%	43.2%	-

[a] The numbers above the diagonal are the root mean square deviations (*rmsds*) in Cα positions between the pairs of enzymes, with the number of matching Cα atoms given in parentheses. The numbers below the diagonal are the pairwise sequence identities.

[b] The enzymes are grouped by their sub-family classification.

Supplementary references

1. Toth M, Vakulenko SB, Smith CA (**2012**). Purification, crystallization and preliminary X-ray analysis of the aminoglycoside-6'-acetyltransferase AAC(6')-Im. **Acta Crystallogr** F68(4): 472-475. doi: 10.1107/S174430910500775X

2. Karplus PA, Diederichs K (**2012**). Linking crystallographic model and data quality. **Science** 336(6084): 1030-1033. doi: 10.1126/science.1218231

3. Chen VB, Arendall WB, Headd JJ, Keedy DA, Immormino RM, Kapral GJ, Murray LW, Richardson JS, Richardson DC (**2010**). MolProbity: All-atom structure validation for macromolecular crystallography. **Acta Crystallogr** D66(1): 12-21. doi: 10.1107/S0907444909042073
